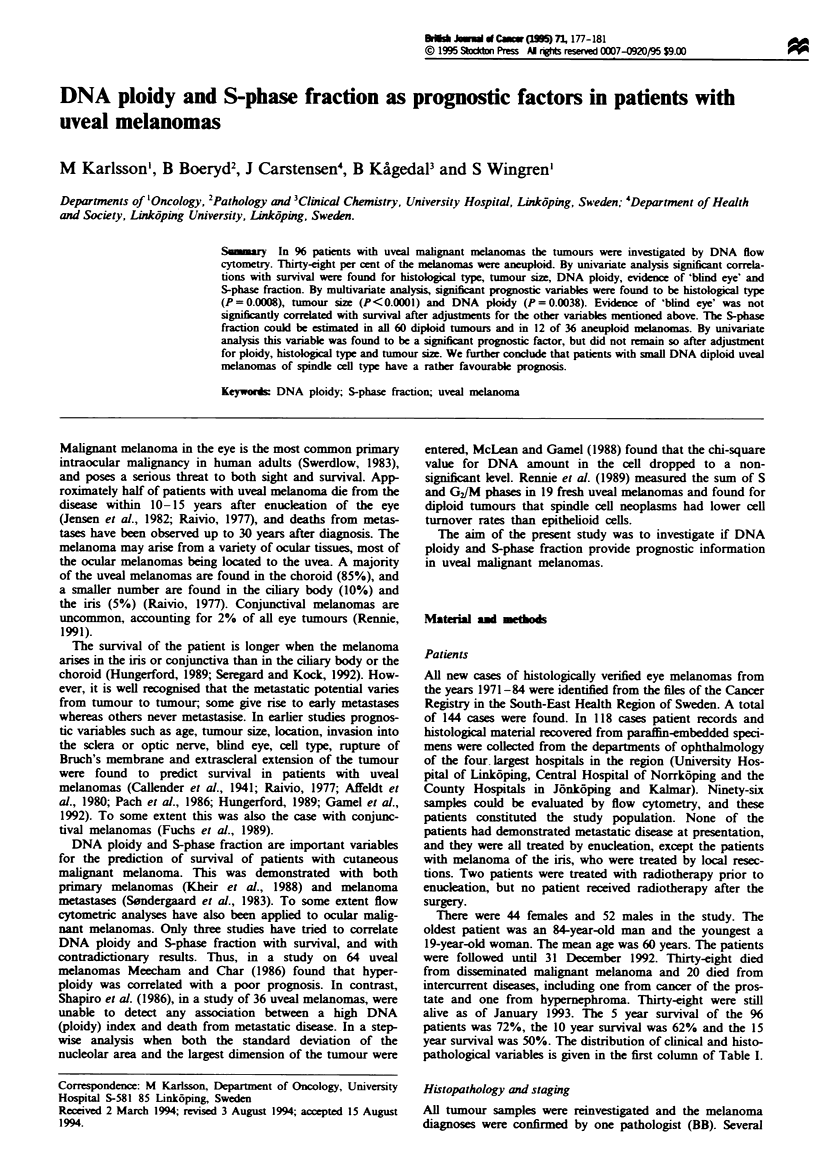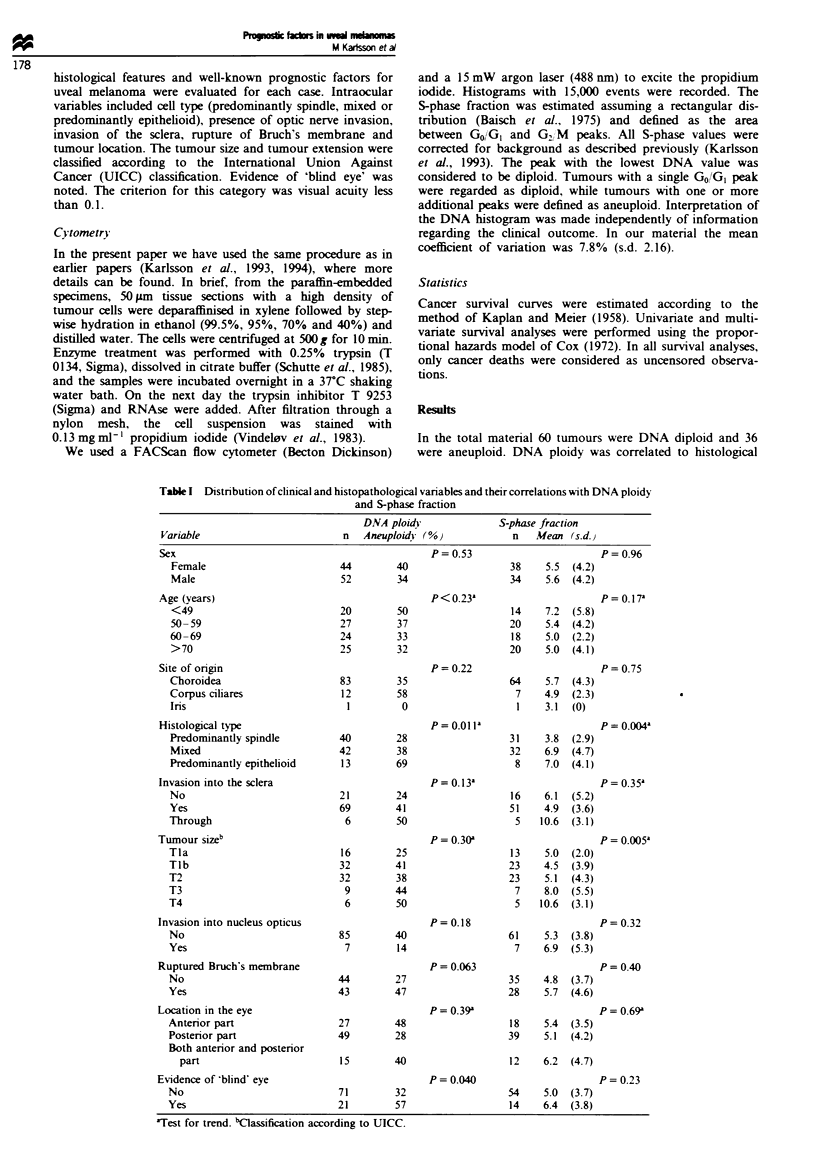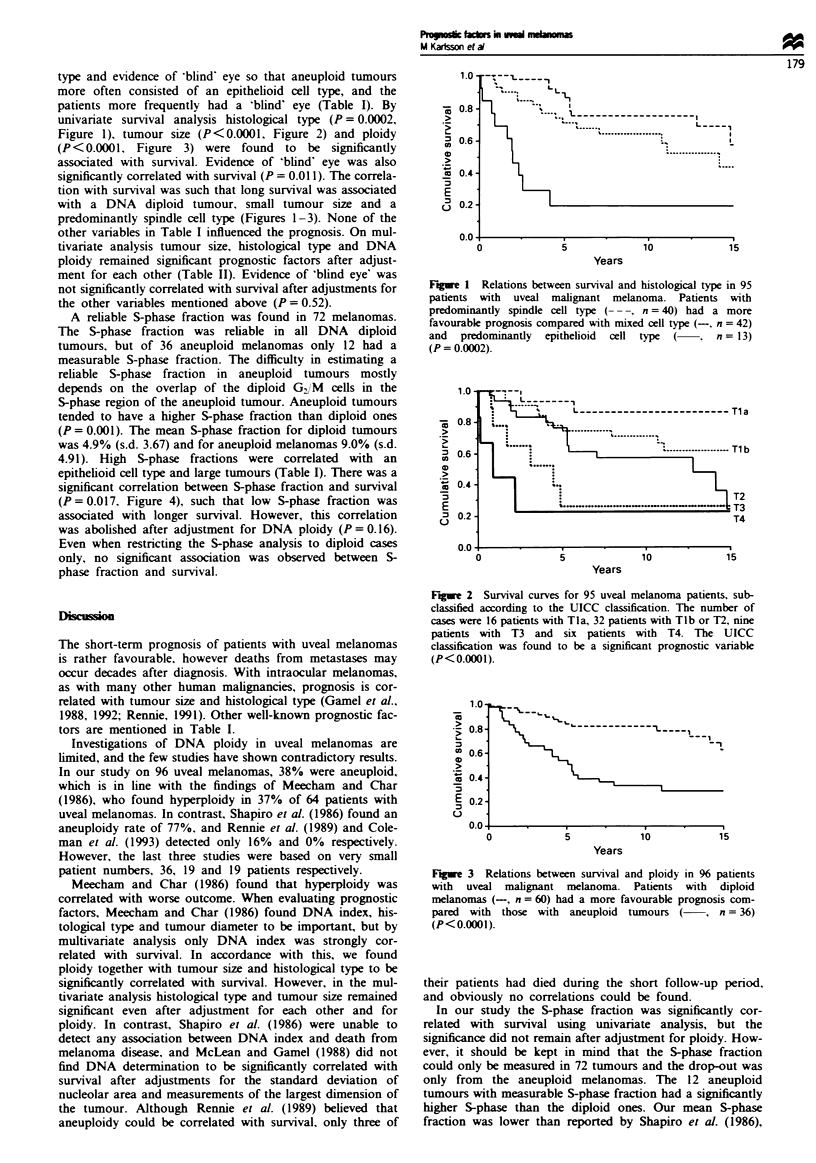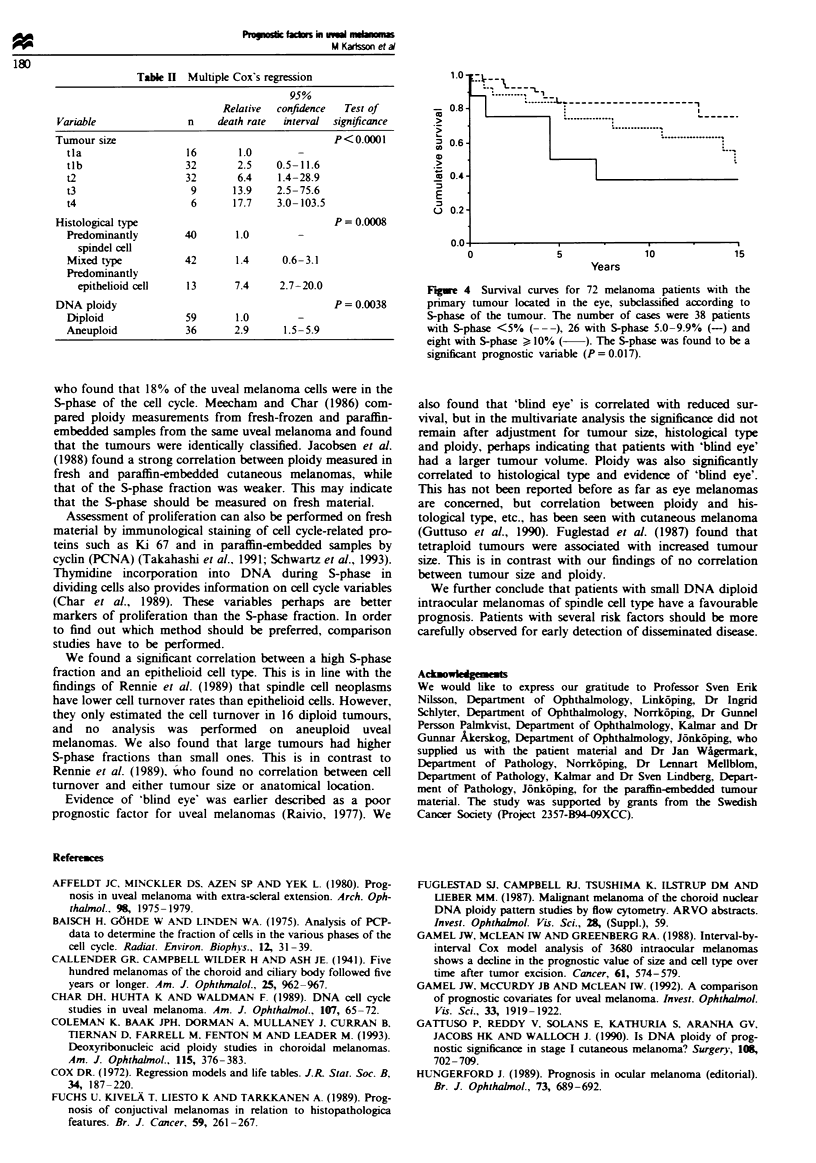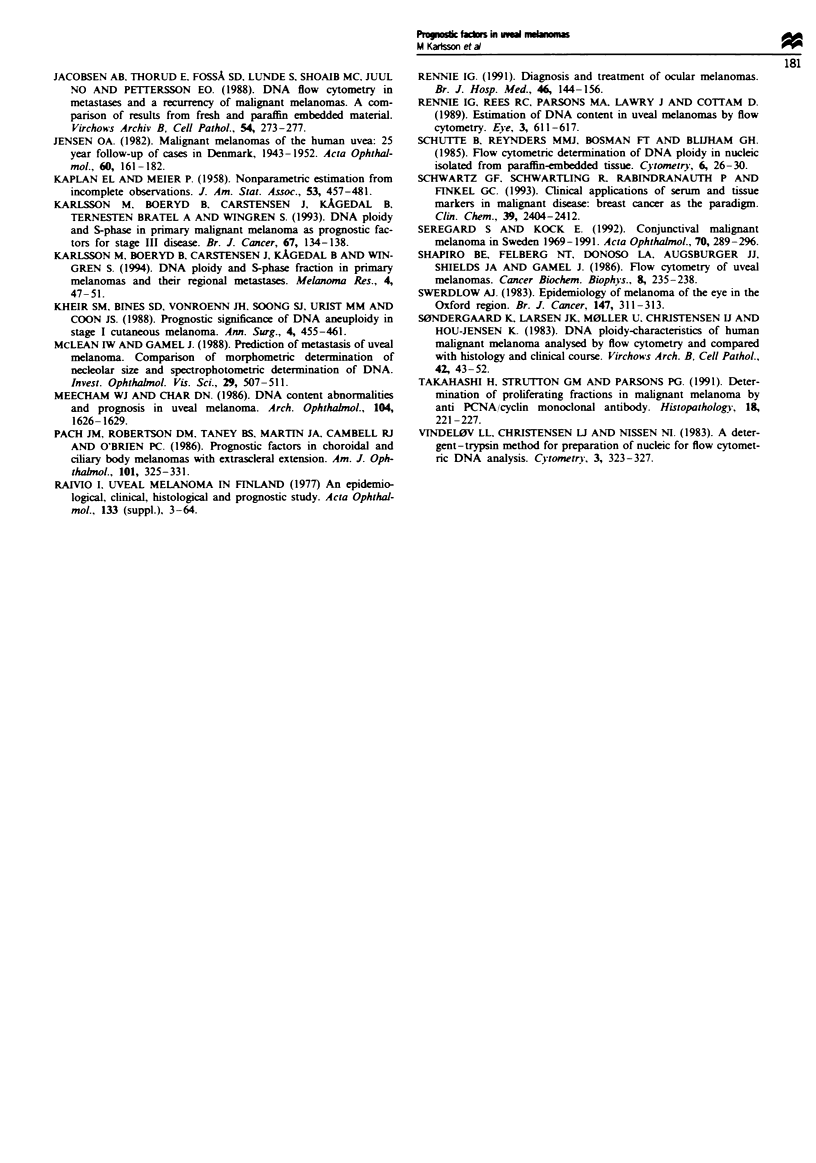# DNA ploidy and S-phase fraction as prognostic factors in patients with uveal melanomas.

**DOI:** 10.1038/bjc.1995.36

**Published:** 1995-01

**Authors:** M. Karlsson, B. Boeryd, J. Carstensen, B. Kågedal, S. Wingren

**Affiliations:** Department of Oncology, University Hospital, Linköping, Sweden.

## Abstract

In 96 patients with uveal malignant melanomas the tumours were investigated by DNA flow cytometry. Thirty-eight per cent of the melanomas were aneuploid. By univariate analysis significant correlations with survival were found for histological type, tumour size, DNA ploidy, evidence of 'blind eye' and S-phase fraction. By multivariate analysis, significant prognostic variables were found to be histological type (P = 0.0008), tumour size (P < 0.0001) and DNA ploidy (P = 0.0038). Evidence of 'blind eye' was not significantly correlated with survival after adjustments for the other variables mentioned above. The S-phase fraction could be estimated in all 60 diploid tumours and in 12 of 36 aneuploid melanomas. By univariate analysis this variable was found to be a significant prognostic factor, but did not remain so after adjustment for ploidy, histological type and tumor size. We further conclude that patients with small DNA diploid uveal melanomas of spindle cell type have a rather favourable prognosis.


© 1995 Stockton Press All rights reserved 0007-0920/95 $9.00

### DNA ploidy and S-phase fraction as prognostic factors in patients with uveal melanomas

M Karlsson[1], B Boeryd[2], J Carstensen[4], B Kågedal[3] and S Wingren[1]

*Departments of [1]Oncology, [2]Pathology and [3]Clinical Chemistry, University Hospital, Linköping, Sweden; [4]Department of Health and Society, Linköping University, Linköping, Sweden.*

**Summary** In 96 patients with uveal malignant melanomas the tumours were investigated by DNA flow cytometry. Thirty-eight per cent of the melanomas were aneuploid. By univariate analysis significant correlations with survival were found for histological type, tumour size, DNA ploidy, evidence of 'blind eye' and S-phase fraction. By multivariate analysis, significant prognostic variables were found to be histological type ($P = 0.0008$), tumour size ($P < 0.0001$) and DNA ploidy ($P = 0.0038$). Evidence of 'blind eye' was not significantly correlated with survival after adjustments for the other variables mentioned above. The S-phase fraction could be estimated in all 60 diploid tumours and in 12 of 36 aneuploid melanomas. By univariate analysis this variable was found to be a significant prognostic factor, but did not remain so after adjustment for ploidy, histological type and tumour size. We further conclude that patients with small DNA diploid uveal melanomas of spindle cell type have a rather favourable prognosis.

**Keywords:** DNA ploidy; S-phase fraction; uveal melanoma

Malignant melanoma in the eye is the most common primary intraocular malignancy in human adults (Swerdlow, 1983), and poses a serious threat to both sight and survival. Approximately half of patients with uveal melanoma die from the disease within 10-15 years after enucleation of the eye (Jensen *et al.*, 1982; Raivio, 1977), and deaths from metastases have been observed up to 30 years after diagnosis. The melanoma may arise from a variety of ocular tissues, most of the ocular melanomas being located to the uvea. A majority of the uveal melanomas are found in the choroid (85%), and a smaller number are found in the ciliary body (10%) and the iris (5%) (Raivio, 1977). Conjunctival melanomas are uncommon, accounting for 2% of all eye tumours (Rennie, 1991).

The survival of the patient is longer when the melanoma arises in the iris or conjunctiva than in the ciliary body or the choroid (Hungerford, 1989; Seregard and Kock, 1992). However, it is well recognised that the metastatic potential varies from tumour to tumour; some give rise to early metastases whereas others never metastasise. In earlier studies prognostic variables such as age, tumour size, location, invasion into the sclera or optic nerve, blind eye, cell type, rupture of Bruch's membrane and extrascleral extension of the tumour were found to predict survival in patients with uveal melanomas (Callender *et al.*, 1941; Raivio, 1977; Affeldt *et al.*, 1980; Pach *et al.*, 1986; Hungerford, 1989; Gamel *et al.*, 1992). To some extent this was also the case with conjunctival melanomas (Fuchs *et al.*, 1989).

DNA ploidy and S-phase fraction are important variables for the prediction of survival of patients with cutaneous malignant melanoma. This was demonstrated with both primary melanomas (Kheir *et al.*, 1988) and melanoma metastases (Søndergaard *et al.*, 1983). To some extent flow cytometric analyses have also been applied to ocular malignant melanomas. Only three studies have tried to correlate DNA ploidy and S-phase fraction with survival, and with contradictionary results. Thus, in a study on 64 uveal melanomas Meecham and Char (1986) found that hyperploidy was correlated with a poor prognosis. In contrast, Shapiro *et al.* (1986), in a study of 36 uveal melanomas, were unable to detect any association between a high DNA (ploidy) index and death from metastatic disease. In a stepwise analysis when both the standard deviation of the nucleolar area and the largest dimension of the tumour were entered, McLean and Gamel (1988) found that the chi-square value for DNA amount in the cell dropped to a non-significant level. Rennie *et al.* (1989) measured the sum of S and $G_2$/M phases in 19 fresh uveal melanomas and found for diploid tumours that spindle cell neoplasms had lower cell turnover rates than epithelioid cells.

The aim of the present study was to investigate if DNA ploidy and S-phase fraction provide prognostic information in uveal malignant melanomas.

### Material and methods

### *Patients*

All new cases of histologically verified eye melanomas from the years 1971-84 were identified from the files of the Cancer Registry in the South-East Health Region of Sweden. A total of 144 cases were found. In 118 cases patient records and histological material recovered from paraffin-embedded specimens were collected from the departments of ophthalmology of the four largest hospitals in the region (University Hospital of Linköping, Central Hospital of Norrköping and the County Hospitals in Jönköping and Kalmar). Ninety-six samples could be evaluated by flow cytometry, and these patients constituted the study population. None of the patients had demonstrated metastatic disease at presentation, and they were all treated by enucleation, except the patients with melanoma of the iris, who were treated by local resections. Two patients were treated with radiotherapy prior to enucleation, but no patient received radiotherapy after the surgery.

There were 44 females and 52 males in the study. The oldest patient was an 84-year-old man and the youngest a 19-year-old woman. The mean age was 60 years. The patients were followed until 31 December 1992. Thirty-eight died from disseminated malignant melanoma and 20 died from intercurrent diseases, including one from cancer of the prostate and one from hypernephroma. Thirty-eight were still alive as of January 1993. The 5 year survival of the 96 patients was 72%, the 10 year survival was 62% and the 15 year survival was 50%. The distribution of clinical and histopathological variables is given in the first column of Table I.

### *Histopathology and staging*

All tumour samples were reinvestigated and the melanoma diagnoses were confirmed by one pathologist (BB). Several

Correspondence: M Karlsson, Department of Oncology, University Hospital S-581 85 Linköping, Sweden
Received 2 March 1994; revised 3 August 1994; accepted 15 August 1994.

histological features and well-known prognostic factors for uveal melanoma were evaluated for each case. Intraocular variables included cell type (predominantly spindle, mixed or predominantly epithelioid), presence of optic nerve invasion, invasion of the sclera, rupture of Bruch's membrane and tumour location. The tumour size and tumour extension were classified according to the International Union Against Cancer (UICC) classification. Evidence of 'blind eye' was noted. The criterion for this category was visual acuity less than 0.1.

### *Cytometry*

In the present paper we have used the same procedure as in earlier papers (Karlsson *et al.*, 1993, 1994), where more details can be found. In brief, from the paraffin-embedded specimens, 50 μm tissue sections with a high density of tumour cells were deparaffinised in xylene followed by step-wise hydration in ethanol (99.5%, 95%, 70% and 40%) and distilled water. The cells were centrifuged at 500 *g* for 10 min. Enzyme treatment was performed with 0.25% trypsin (T 0134, Sigma), dissolved in citrate buffer (Schutte *et al.*, 1985), and the samples were incubated overnight in a 37°C shaking water bath. On the next day the trypsin inhibitor T 9253 (Sigma) and RNAse were added. After filtration through a nylon mesh, the cell suspension was stained with 0.13 mg $ml^{-1}$ propidium iodide (Vindeløv *et al.*, 1983).

We used a FACScan flow cytometer (Becton Dickinson) and a 15 mW argon laser (488 nm) to excite the propidium iodide. Histograms with 15,000 events were recorded. The S-phase fraction was estimated assuming a rectangular distribution (Baisch *et al.*, 1975) and defined as the area between $G_0/G_1$ and $G_2/M$ peaks. All S-phase values were corrected for background as described previously (Karlsson *et al.*, 1993). The peak with the lowest DNA value was considered to be diploid. Tumours with a single $G_0/G_1$ peak were regarded as diploid, while tumours with one or more additional peaks were defined as aneuploid. Interpretation of the DNA histogram was made independently of information regarding the clinical outcome. In our material the mean coefficient of variation was 7.8% (s.d. 2.16).

### *Statistics*

Cancer survival curves were estimated according to the method of Kaplan and Meier (1958). Univariate and multivariate survival analyses were performed using the proportional hazards model of Cox (1972). In all survival analyses, only cancer deaths were considered as uncensored observations.

### Results

In the total material 60 tumours were DNA diploid and 36 were aneuploid. DNA ploidy was correlated to histological

**Table I** Distribution of clinical and histopathological variables and their correlations with DNA ploidy and S-phase fraction

| *Variable* | n | *DNA ploidy* *Aneuploidy (%)* | | n | *S-phase fraction* *Mean* | *(s.d.)* | |
|---|---|---|---|---|---|---|---|
| Sex | | | $P = 0.53$ | | | | $P = 0.96$ |
| Female | 44 | 40 | | 38 | 5.5 | (4.2) | |
| Male | 52 | 34 | | 34 | 5.6 | (4.2) | |
| Age (years) | | | $P < 0.23$[a] | | | | $P = 0.17$[a] |
| <49 | 20 | 50 | | 14 | 7.2 | (5.8) | |
| 50–59 | 27 | 37 | | 20 | 5.4 | (4.2) | |
| 60–69 | 24 | 33 | | 18 | 5.0 | (2.2) | |
| >70 | 25 | 32 | | 20 | 5.0 | (4.1) | |
| Site of origin | | | $P = 0.22$ | | | | $P = 0.75$ |
| Choroidea | 83 | 35 | | 64 | 5.7 | (4.3) | |
| Corpus ciliares | 12 | 58 | | 7 | 4.9 | (2.3) | |
| Iris | 1 | 0 | | 1 | 3.1 | (0) | |
| Histological type | | | $P = 0.011$[a] | | | | $P = 0.004$[a] |
| Predominantly spindle | 40 | 28 | | 31 | 3.8 | (2.9) | |
| Mixed | 42 | 38 | | 32 | 6.9 | (4.7) | |
| Predominantly epithelioid | 13 | 69 | | 8 | 7.0 | (4.1) | |
| Invasion into the sclera | | | $P = 0.13$[a] | | | | $P = 0.35$[a] |
| No | 21 | 24 | | 16 | 6.1 | (5.2) | |
| Yes | 69 | 41 | | 51 | 4.9 | (3.6) | |
| Through | 6 | 50 | | 5 | 10.6 | (3.1) | |
| Tumour size[b] | | | $P = 0.30$[a] | | | | $P = 0.005$[a] |
| T1a | 16 | 25 | | 13 | 5.0 | (2.0) | |
| T1b | 32 | 41 | | 23 | 4.5 | (3.9) | |
| T2 | 32 | 38 | | 23 | 5.1 | (4.3) | |
| T3 | 9 | 44 | | 7 | 8.0 | (5.5) | |
| T4 | 6 | 50 | | 5 | 10.6 | (3.1) | |
| Invasion into nucleus opticus | | | $P = 0.18$ | | | | $P = 0.32$ |
| No | 85 | 40 | | 61 | 5.3 | (3.8) | |
| Yes | 7 | 14 | | 7 | 6.9 | (5.3) | |
| Ruptured Bruch's membrane | | | $P = 0.063$ | | | | $P = 0.40$ |
| No | 44 | 27 | | 35 | 4.8 | (3.7) | |
| Yes | 43 | 47 | | 28 | 5.7 | (4.6) | |
| Location in the eye | | | $P = 0.39$[a] | | | | $P = 0.69$[a] |
| Anterior part | 27 | 48 | | 18 | 5.4 | (3.5) | |
| Posterior part | 49 | 28 | | 39 | 5.1 | (4.2) | |
| Both anterior and posterior part | 15 | 40 | | 12 | 6.2 | (4.7) | |
| Evidence of 'blind' eye | | | $P = 0.040$ | | | | $P = 0.23$ |
| No | 71 | 32 | | 54 | 5.0 | (3.7) | |
| Yes | 21 | 57 | | 14 | 6.4 | (3.8) | |

[a]Test for trend. [b]Classification according to UICC.

type and evidence of 'blind' eye so that aneuploid tumours more often consisted of an epithelioid cell type, and the patients more frequently had a 'blind' eye (Table I). By univariate survival analysis histological type ($P = 0.0002$, Figure 1), tumour size ($P < 0.0001$, Figure 2) and ploidy ($P < 0.0001$, Figure 3) were found to be significantly associated with survival. Evidence of 'blind' eye was also significantly correlated with survival ($P = 0.011$). The correlation with survival was such that long survival was associated with a DNA diploid tumour, small tumour size and a predominantly spindle cell type (Figures 1-3). None of the other variables in Table I influenced the prognosis. On multivariate analysis tumour size, histological type and DNA ploidy remained significant prognostic factors after adjustment for each other (Table II). Evidence of 'blind eye' was not significantly correlated with survival after adjustments for the other variables mentioned above ($P = 0.52$).

A reliable S-phase fraction was found in 72 melanomas. The S-phase fraction was reliable in all DNA diploid tumours, but of 36 aneuploid melanomas only 12 had a measurable S-phase fraction. The difficulty in estimating a reliable S-phase fraction in aneuploid tumours mostly depends on the overlap of the diploid $G_2/M$ cells in the S-phase region of the aneuploid tumour. Aneuploid tumours tended to have a higher S-phase fraction than diploid ones ($P = 0.001$). The mean S-phase fraction for diploid tumours was 4.9% (s.d. 3.67) and for aneuploid melanomas 9.0% (s.d. 4.91). High S-phase fractions were correlated with an epithelioid cell type and large tumours (Table I). There was a significant correlation between S-phase fraction and survival ($P = 0.017$, Figure 4), such that low S-phase fraction was associated with longer survival. However, this correlation was abolished after adjustment for DNA ploidy ($P = 0.16$). Even when restricting the S-phase analysis to diploid cases only, no significant association was observed between S-phase fraction and survival.

### Discussion

The short-term prognosis of patients with uveal melanomas is rather favourable, however deaths from metastases may occur decades after diagnosis. With intraocular melanomas, as with many other human malignancies, prognosis is correlated with tumour size and histological type (Gamel *et al.*, 1988, 1992; Rennie, 1991). Other well-known prognostic factors are mentioned in Table I.

Investigations of DNA ploidy in uveal melanomas are limited, and the few studies have shown contradictory results. In our study on 96 uveal melanomas, 38% were aneuploid, which is in line with the findings of Meecham and Char (1986), who found hyperploidy in 37% of 64 patients with uveal melanomas. In contrast, Shapiro *et al.* (1986) found an aneuploidy rate of 77%, and Rennie *et al.* (1989) and Coleman *et al.* (1993) detected only 16% and 0% respectively. However, the last three studies were based on very small patient numbers, 36, 19 and 19 patients respectively.

Meecham and Char (1986) found that hyperploidy was correlated with worse outcome. When evaluating prognostic factors, Meecham and Char (1986) found DNA index, histological type and tumour diameter to be important, but by multivariate analysis only DNA index was strongly correlated with survival. In accordance with this, we found ploidy together with tumour size and histological type to be significantly correlated with survival. However, in the multivariate analysis histological type and tumour size remained significant even after adjustment for each other and for ploidy. In contrast, Shapiro *et al.* (1986) were unable to detect any association between DNA index and death from melanoma disease, and McLean and Gamel (1988) did not find DNA determination to be significantly correlated with survival after adjustments for the standard deviation of nucleolar area and measurements of the largest dimension of the tumour. Although Rennie *et al.* (1989) believed that aneuploidy could be correlated with survival, only three of their patients had died during the short follow-up period, and obviously no correlations could be found.

In our study the S-phase fraction was significantly correlated with survival using univariate analysis, but the significance did not remain after adjustment for ploidy. However, it should be kept in mind that the S-phase fraction could only be measured in 72 tumours and the drop-out was only from the aneuploid melanomas. The 12 aneuploid tumours with measurable S-phase fraction had a significantly higher S-phase than the diploid ones. Our mean S-phase fraction was lower than reported by Shapiro *et al.* (1986),

**Figure 1** Relations between survival and histological type in 95 patients with uveal malignant melanoma. Patients with predominantly spindle cell type (- - -, $n = 40$) had a more favourable prognosis compared with mixed cell type (—, $n = 42$) and predominantly epithelioid cell type (——, $n = 13$) ($P = 0.0002$).

**Figure 2** Survival curves for 95 uveal melanoma patients, subclassified according to the UICC classification. The number of cases were 16 patients with T1a, 32 patients with T1b or T2, nine patients with T3 and six patients with T4. The UICC classification was found to be a significant prognostic variable ($P < 0.0001$).

**Figure 3** Relations between survival and ploidy in 96 patients with uveal malignant melanoma. Patients with diploid melanomas (—, $n = 60$) had a more favourable prognosis compared with those with aneuploid tumours (——, $n = 36$) ($P < 0.0001$).

**Table II** Multiple Cox's regression

| Variable | n | Relative death rate | 95% confidence interval | Test of significance |
|---|---|---|---|---|
| Tumour size | | | | $P<0.0001$ |
| t1a | 16 | 1.0 | – | |
| t1b | 32 | 2.5 | 0.5–11.6 | |
| t2 | 32 | 6.4 | 1.4–28.9 | |
| t3 | 9 | 13.9 | 2.5–75.6 | |
| t4 | 6 | 17.7 | 3.0–103.5 | |
| Histological type | | | | $P=0.0008$ |
| Predominantly spindel cell | 40 | 1.0 | – | |
| Mixed type | 42 | 1.4 | 0.6–3.1 | |
| Predominantly epithelioid cell | 13 | 7.4 | 2.7–20.0 | |
| DNA ploidy | | | | $P=0.0038$ |
| Diploid | 59 | 1.0 | – | |
| Aneuploid | 36 | 2.9 | 1.5–5.9 | |

**Figure 4** Survival curves for 72 melanoma patients with the primary tumour located in the eye, subclassified according to S-phase of the tumour. The number of cases were 38 patients with S-phase <5% (- - -), 26 with S-phase 5.0–9.9% (—) and eight with S-phase ≥10% (——). The S-phase was found to be a significant prognostic variable ($P=0.017$).

who found that 18% of the uveal melanoma cells were in the S-phase of the cell cycle. Meecham and Char (1986) compared ploidy measurements from fresh-frozen and paraffin-embedded samples from the same uveal melanoma and found that the tumours were identically classified. Jacobsen *et al.* (1988) found a strong correlation between ploidy measured in fresh and paraffin-embedded cutaneous melanomas, while that of the S-phase fraction was weaker. This may indicate that the S-phase should be measured on fresh material.

Assessment of proliferation can also be performed on fresh material by immunological staining of cell cycle-related proteins such as Ki 67 and in paraffin-embedded samples by cyclin (PCNA) (Takahashi *et al.*, 1991; Schwartz *et al.*, 1993). Thymidine incorporation into DNA during S-phase in dividing cells also provides information on cell cycle variables (Char *et al.*, 1989). These variables perhaps are better markers of proliferation than the S-phase fraction. In order to find out which method should be preferred, comparison studies have to be performed.

We found a significant correlation between a high S-phase fraction and an epithelioid cell type. This is in line with the findings of Rennie *et al.* (1989) that spindle cell neoplasms have lower cell turnover rates than epithelioid cells. However, they only estimated the cell turnover in 16 diploid tumours, and no analysis was performed on aneuploid uveal melanomas. We also found that large tumours had higher S-phase fractions than small ones. This is in contrast to Rennie *et al.* (1989), who found no correlation between cell turnover and either tumour size or anatomical location.

Evidence of 'blind eye' was earlier described as a poor prognostic factor for uveal melanomas (Raivio, 1977). We also found that 'blind eye' is correlated with reduced survival, but in the multivariate analysis the significance did not remain after adjustment for tumour size, histological type and ploidy, perhaps indicating that patients with 'blind eye' had a larger tumour volume. Ploidy was also significantly correlated to histological type and evidence of 'blind eye'. This has not been reported before as far as eye melanomas are concerned, but correlation between ploidy and histological type, etc., has been seen with cutaneous melanoma (Guttuso *et al.*, 1990). Fuglestad *et al.* (1987) found that tetraploid tumours were associated with increased tumour size. This is in contrast with our findings of no correlation between tumour size and ploidy.

We further conclude that patients with small DNA diploid intraocular melanomas of spindle cell type have a favourable prognosis. Patients with several risk factors should be more carefully observed for early detection of disseminated disease.

**Acknowledgements**

We would like to express our gratitude to Professor Sven Erik Nilsson, Department of Ophthalmology, Linköping, Dr Ingrid Schlyter, Department of Ophthalmology, Norrköping, Dr Gunnel Persson Palmkvist, Department of Ophthalmology, Kalmar and Dr Gunnar Åkerskog, Department of Ophthalmology, Jönköping, who supplied us with the patient material and Dr Jan Wågermark, Department of Pathology, Norrköping, Dr Lennart Mellblom, Department of Pathology, Kalmar and Dr Sven Lindberg, Department of Pathology, Jönköping, for the paraffin-embedded tumour material. The study was supported by grants from the Swedish Cancer Society (Project 2357-B94-09XCC).

**References**

AFFELDT JC, MINCKLER DS, AZEN SP AND YEK L. (1980). Prognosis in uveal melanoma with extra-scleral extension. *Arch. Ophthalmol.*, **98**, 1975–1979.

BAISCH H, GÖHDE W AND LINDEN WA. (1975). Analysis of PCP-data to determine the fraction of cells in the various phases of the cell cycle. *Radiat. Environ. Biophys.*, **12**, 31–39.

CALLENDER GR, CAMPBELL WILDER H AND ASH JE. (1941). Five hundred melanomas of the choroid and ciliary body followed five years or longer. *Am. J. Ophthmalol.*, **25**, 962–967.

CHAR DH, HUHTA K AND WALDMAN F. (1989). DNA cell cycle studies in uveal melanoma. *Am. J. Ophthalmol.*, **107**, 65–72.

COLEMAN K, BAAK JPH, DORMAN A, MULLANEY J, CURRAN B, TIERNAN D, FARRELL M, FENTON M AND LEADER M. (1993). Deoxyribonucleic acid ploidy studies in choroidal melanomas. *Am. J. Ophthalmol.*, **115**, 376–383.

COX DR. (1972). Regression models and life tables. *J.R. Stat. Soc. B*, **34**, 187–220.

FUCHS U, KIVELÄ T, LIESTO K AND TARKKANEN A. (1989). Prognosis of conjuctival melanomas in relation to histopathologica features. *Br. J. Cancer*, **59**, 261–267.

FUGLESTAD SJ, CAMPBELL RJ, TSUSHIMA K, ILSTRUP DM AND LIEBER MM. (1987). Malignant melanoma of the choroid nuclear DNA ploidy pattern studies by flow cytometry. ARVO abstracts. *Invest. Ophthalmol. Vis. Sci.*, **28**, (Suppl.), 59.

GAMEL JW, McLEAN IW AND GREENBERG RA. (1988). Interval-by-interval Cox model analysis of 3680 intraocular melanomas shows a decline in the prognostic value of size and cell type over time after tumor excision. *Cancer*, **61**, 574–579.

GAMEL JW, McCURDY JB AND McLEAN IW. (1992). A comparison of prognostic covariates for uveal melanoma. *Invest. Ophthalmol. Vis. Sci.*, **33**, 1919–1922.

GATTUSO P, REDDY V, SOLANS E, KATHURIA S, ARANHA GV, JACOBS HK AND WALLOCH J. (1990). Is DNA ploidy of prognostic significance in stage I cutaneous melanoma? *Surgery*, **108**, 702–709.

HUNGERFORD J. (1989). Prognosis in ocular melanoma (editorial). *Br. J. Ophthalmol.*, **73**, 689–692.

JACOBSEN AB, THORUD E, FOSSÅ SD, LUNDE S, SHOAIB MC, JUUL NO AND PETTERSSON EO. (1988). DNA flow cytometry in metastases and a recurrency of malignant melanomas. A comparison of results from fresh and paraffin embedded material. *Virchows Archiv B, Cell Pathol.*, **54**, 273–277.

JENSEN OA. (1982). Malignant melanomas of the human uvea: 25 year follow-up of cases in Denmark, 1943–1952. *Acta Ophthalmol.*, **60**, 161–182.

KAPLAN EL AND MEIER P. (1958). Nonparametric estimation from incomplete observations. *J. Am. Stat. Assoc.*, **53**, 457–481.

KARLSSON M, BOERYD B, CARSTENSEN J, KÅGEDAL B, TERNESTEN BRATEL A AND WINGREN S. (1993). DNA ploidy and S-phase in primary malignant melanoma as prognostic factors for stage III disease. *Br. J. Cancer*, **67**, 134–138.

KARLSSON M, BOERYD B, CARSTENSEN J, KÅGEDAL B AND WINGREN S. (1994). DNA ploidy and S-phase fraction in primary melanomas and their regional metastases. *Melanoma Res.*, **4**, 47–51.

KHEIR SM, BINES SD, VONROENN JH, SOONG SJ, URIST MM AND COON JS. (1988). Prognostic significance of DNA aneuploidy in stage I cutaneous melanoma. *Ann. Surg.*, **4**, 455–461.

MCLEAN IW AND GAMEL J. (1988). Prediction of metastasis of uveal melanoma. Comparison of morphometric determination of necleolar size and spectrophotometric determination of DNA. *Invest. Ophthalmol. Vis. Sci.*, **29**, 507–511.

MEECHAM WJ AND CHAR DN. (1986). DNA content abnormalities and prognosis in uveal melanoma. *Arch. Ophthalmol.*, **104**, 1626–1629.

PACH JM, ROBERTSON DM, TANEY BS, MARTIN JA, CAMBELL RJ AND O'BRIEN PC. (1986). Prognostic factors in choroidal and ciliary body melanomas with extrascleral extension. *Am. J. Ophthalmol.*, **101**, 325–331.

RAIVIO I, UVEAL MELANOMA IN FINLAND (1977) An epidemiological, clinical, histological and prognostic study. *Acta Ophthalmol.*, **133** (suppl.), 3–64.

RENNIE IG. (1991). Diagnosis and treatment of ocular melanomas. *Br. J. Hosp. Med.*, **46**, 144–156.

RENNIE IG, REES RC, PARSONS MA, LAWRY J AND COTTAM D. (1989). Estimation of DNA content in uveal melanomas by flow cytometry. *Eye*, **3**, 611–617.

SCHUTTE B, REYNDERS MMJ, BOSMAN FT AND BLIJHAM GH. (1985). Flow cytometric determination of DNA ploidy in nucleic isolated from paraffin-embedded tissue. *Cytometry*, **6**, 26–30.

SCHWARTZ GF, SCHWARTLING R, RABINDRANAUTH P AND FINKEL GC. (1993). Clinical applications of serum and tissue markers in malignant disease: breast cancer as the paradigm. *Clin. Chem.*, **39**, 2404–2412.

SEREGARD S AND KOCK E. (1992). Conjunctival malignant melanoma in Sweden 1969–1991. *Acta Ophthalmol.*, **70**, 289–296.

SHAPIRO BE, FELBERG NT, DONOSO LA, AUGSBURGER JJ, SHIELDS JA AND GAMEL J. (1986). Flow cytometry of uveal melanomas. *Cancer Biochem. Biophys.*, **8**, 235–238.

SWERDLOW AJ. (1983). Epidemiology of melanoma of the eye in the Oxford region. *Br. J. Cancer*, **147**, 311–313.

SØNDERGAARD K, LARSEN JK, MØLLER U, CHRISTENSEN IJ AND HOU-JENSEN K. (1983). DNA ploidy-characteristics of human malignant melanoma analysed by flow cytometry and compared with histology and clinical course. *Virchows Arch. B, Cell Pathol.*, **42**, 43–52.

TAKAHASHI H, STRUTTON GM AND PARSONS PG. (1991). Determination of proliferating fractions in malignant melanoma by anti PCNA/cyclin monoclonal antibody. *Histopathology*, **18**, 221–227.

VINDELØV LL, CHRISTENSEN IJ AND NISSEN NI. (1983). A detergent–trypsin method for preparation of nucleic for flow cytometric DNA analysis. *Cytometry*, **3**, 323–327.